# The First Korean Case with Cardiac, Facial, and Digital Anomalies with Developmental Delay Caused by De Novo *TRAF7* p.Arg655Gln Variant

**DOI:** 10.3390/ijms25073701

**Published:** 2024-03-26

**Authors:** Kyung Hee Kim, Ji Yoon Han, Joonhong Park, Jung Sun Cho

**Affiliations:** 1 Division of Cardiology, Department of Internal Medicine, College of Medicine, The Catholic University of Korea, Seoul 06591, Republic of Korea; kyunghee.kim93@gmail.com; 2 Department of Pediatrics, College of Medicine, The Catholic University of Korea, Seoul 06591, Republic of Korea; han024@catholic.ac.kr; 3 Department of Laboratory Medicine, Jeonbuk National University Medical School and Hospital, Jeonju 54907, Republic of Korea; miziro@jbnu.ac.kr; 4 Research Institute of Clinical Medicine of Jeonbuk National University, Biomedical Research Institute of Jeonbuk National University Hospital, Jeonju 54907, Republic of Korea; 5 Catholic Research Institute for Intractable Cardiovascular Disease, College of Medicine, The Catholic University of Korea, Seoul 06591, Republic of Korea

**Keywords:** CAFDADD syndrome, blepharophimosis-mental retardation syndromes, *TRAF7*, p.Arg655Gln, clinical exome sequencing

## Abstract

*TRAF7*-related disorders represent some of the rarest inherited disorders, exhibiting clinical features that overlap with cardiac, facial, and digital anomalies with developmental delay (CAFDADD) syndrome, as well as blepharophimosis-mental retardation syndrome (BMRS). A 36-year-old male, presenting with total blindness, blepharophimosis, and intellectual disability, was admitted for the assessment of resting dyspnea several months previously. He had a history of being diagnosed with obstructive sleep apnea (OSA). Transesophageal and transthoracic echocardiography unveiled right ventricular dilatation without significant pulmonary hypertension, bicuspid aortic valve with aortic root aneurysm, and aortic regurgitation in the proband. Sanger sequencing identified a de novo *TRAF7* variant (c.1964G>A; p.Arg655Gln). Subsequently, aortic root replacement using the Bentall procedure was performed. However, despite the surgery, he continued to experience dyspnea. Upon re-evaluating OSA with polysomnography, it was discovered that continuous positive airway pressure support alleviated his symptoms. The underlying cause of his symptoms was attributed to OSA, likely exacerbated by the vertebral anomaly and short neck associated with CAFDADD syndrome. Clinicians should be attentive to the symptoms associated with OSA as it is a potentially serious medical condition in patients with *TRAF7* variants.

## 1. Introduction

The cardiac, facial, and digital anomalies with developmental delay (CAFDADD) syndrome is a developmental disorder that affects multiple systems, characterized by diverse cardiac and digital anomalies, along with facial dysmorphism. Some individuals with this condition may also experience seizures and exhibit ocular or aural abnormalities [1,2]. CAFDADD syndrome is exceptionally rare, and it shares clinical features with blepharophimosis-mental retardation syndrome (BMRS). The syndrome is caused by germline missense mutations in tumor necrosis factor receptor-associated factor 7 (*TRAF7*) [2]. *TRAF7* is a member of the versatile *TRAF* family and plays a role in various biological processes, including embryogenic development, tissue homeostasis, humoral immune response, ubiquitination, and myogenesis [3]. Additionally, *TRAF7* acts as a mediator in the MAP kinase and NF-kB signaling pathways. Somatic mutations in *TRAF7* have been observed in meningioma and mesothelioma. CAFDADD syndrome’s full phenotypic spectrum is not well understood due to its rarity, but a recent large-scale analysis reported the clinical and mutational spectrum of CAFDADD syndrome [4]. The common features of CAFDADD syndrome include a recognizable facial gestalt (particularly blepharophimosis), digital anomalies, short neck, pectus carinatum, patent ductus arteriosus (PDA), and developmental delays [4,5,6]. However, most investigated patients with *TRAF7* mutations have been pediatric, and long-term clinical findings for patients with CAFDADD syndrome remain unknown. To date, approximately 20 missense variants associated with CAFDADD have been reported as either pathogenic or likely pathogenic, while no other types of variants or variations have been identified (https://www.ncbi.nlm.nih.gov/clinvar; accessed on 10 March 2024).

In this report, we present the case of a 36-year-old man diagnosed with CAFDADD syndrome, who also exhibited heart failure, associated obstructive sleep apnea, bicuspid aortic valve disease, and aortic aneurysm. Notably, this report marks the first documented case of CAFDADD syndrome in the Republic of Korea attributed to a de novo *TRAF7* p.Arg655Gln variant.

## 2. Case Presentation

**Figure 1:**
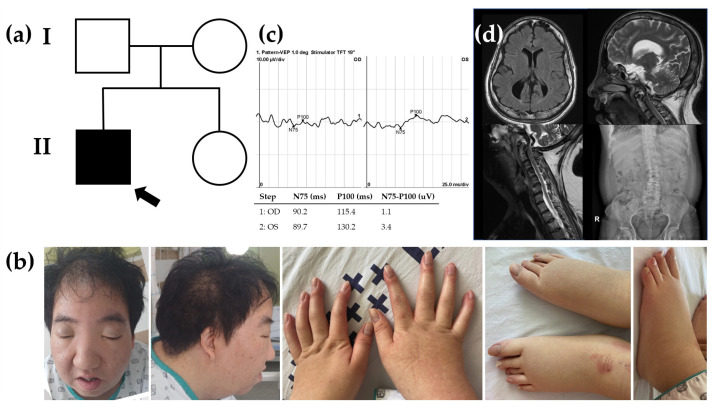
Pedigree, clinical phenotypes, and radiologic findings. (**a**) Pedigree of the proband (arrow) carrying a heterozygous *TRAF7* variant in an autosomal dominant inheritance and his family members. (**b**) Short stature (153.7 cm), short neck, humped back, excess nuchal skin, facial dysmorphism (swollen eyelids, blepharophimosis), arachnodactyly, and edema in both lower extremities. (**c**) Visual evoked potentials recorded in 2-year-old showing small amplitude VEP in both eyes. (**d**) Diffuse atrophy and skull bone thickening on brain magnetic resonance imaging (MRI). On cervical MRI, severe angulation of the craniocervical junction and spinal cord compression. L-spine scoliosis.

**Figure 2:**
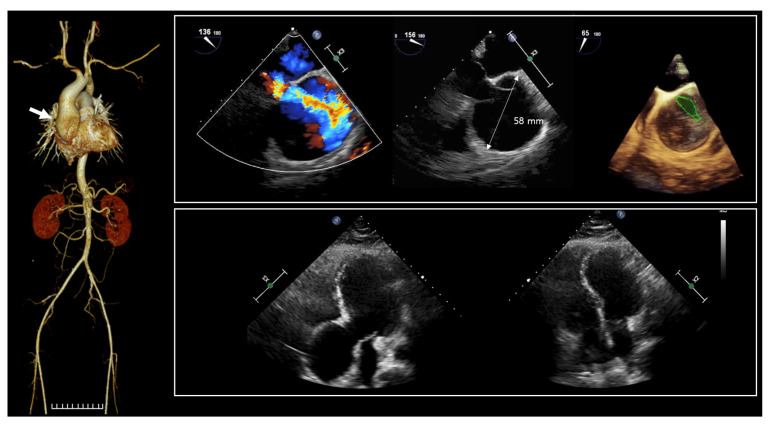
Computed tomography scan of the chest aorta revealed aneurysmal dilatation of about 5.8 cm from the aortic annulus to the proximal ascending aorta (arrow). Color doppler echocardiography of aortic root aneurysm; red and blue represents flow towards and away from the transducer, respectively. A 3-dimensional transesophageal echocardiogram shows aortic root aneurysm and bicuspid AV (sinus of Valsalva = 58 mm) (upper panel). Bicuspid AV is represented by green outline. Transthoracic echocardiography at present admission showed dilatation of aortic root and RV dilatation (lower panel).

The proband (II-1 in Figure 1a), a 36-year-old man, was admitted to the hospital due to dyspnea. Since birth, he has experienced hypotonia, blindness in both eyes, and hearing impairment. He was the first child of nonconsanguineous parents, and the pregnancy was uneventful at 38 weeks of gestation. Family history was unremarkable. His growth percentiles were within normal ranges at birth. In the early infant period, he showed generalized hypotonia, and growth motor slowly improved in childhood. Throughout his early development, he displayed global developmental delay, eventually reaching borderline intellectual disability (ID) with an intellectual quotient of 75 using the Wechsler Adult Intelligence Scale. As a result, he attended a specialized school for handicapped children due to total blindness and a borderline IQ. He did not live independently and required assistance for daily living. Moreover, he encountered challenges in obtaining employment. Snoring has been a consistent issue since childhood, leading to a diagnosis of obstructive sleep apnea based on polysomnogram results. His body mass index was 28 kg/m^2^ (90th percentile), which corresponds to overweight. Upon admission, vital signs were stable, with blood pressure at 110/60 mmHg, heart rate at 60 beats per minute, respiratory rate at 16 beats per minute, and body temperature at 36.6 °C. Physical examination revealed several abnormalities, including short stature (153.7 cm, below the 3rd percentile), a short neck, pectus carinatum, humped back, excess nuchal skin, facial dysmorphism (swollen eyelids, blepharophimosis, wide nasal bridge, a high forehead), vertebral anomaly, arachnodactyly, and edema in both lower extremities (Figure 1b). He was diagnosed with almost total blindness with nonlight perception at around 2 years old, and an ophthalmologic examination revealed optic atrophy (Figure 1c). Brain magnetic resonance imaging indicated diffuse cerebral atrophy, and cervical computed tomography scan revealed disc bulging with spondylosis (Figure 1d). Laboratory findings showed an elevated N-terminal prohormone of brain natriuretic peptide (248.10 pg/mL; reference range, 42.5 to 106.4), while highly sensitive troponin T (0.004 ng/mL; reference range, <0.014) and creatine kinase-MB (0.90 ng/mL; reference range, <2.2) were within normal limits. Nonspecific findings were observed in complete blood count, blood chemistry, metabolic screening, and urinalysis. Two years prior to admission, a transthoracic echocardiogram (TTE) conducted at the outpatient setting displayed normal left ventricular systolic function with an ejection fraction (EF) of 59%, and the ascending aorta measured 45 mm in diameter. Due to poor echo windows caused by pectus carinatum, assessment of the aortic valve and aortic root was inconclusive. A chest aorta computer tomography scan (CT) performed during the present admission revealed marked aneurysmal dilatation, approximately 5.8 cm from the aortic annulus to the proximal ascending aorta (Figure 2). A three-dimensional transesophageal echocardiogram exhibited aortic root aneurysm and bicuspid aortic valve (sinus of Valsalva = 58 mm) (upper panel in Figure 2). TTE during the current admission showed dilatation of the aortic root and right ventricular dilatation (lower panel in Figure 2).

**Figure 3:**
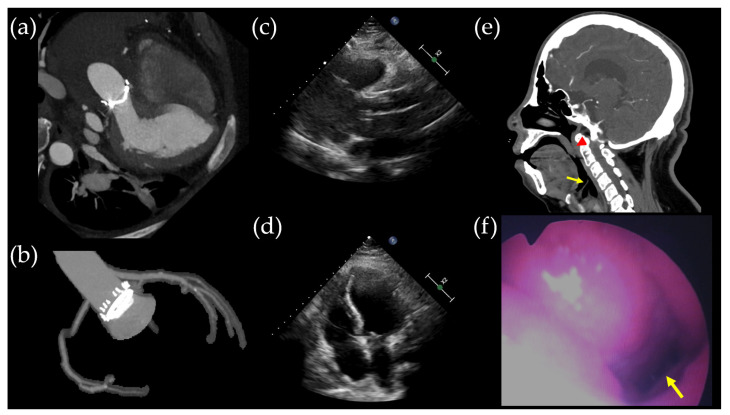
Postoperative radiologic findings. (**a**,**b**) Coronary artery computed tomography performed on postoperative day (POD) 32 illustrates the successful Bentall operation, which included aortic valve replacement (21 mm), ascending aorta replacement, and coronary button implantation. (**c**,**d**) Transthoracic echocardiogram conducted on POD 32 revealed unchanged right ventricular dilatation in size compared to the preoperative transthoracic echocardiogram. Facial CT scan (**e**) and laryngoscopy (**f**) identified narrowing of the nasopharynx and oropharynx due to a retropositioned mandible, with the epiglottis indicated by the yellow arrow and the uvula by the red arrowhead.

The patient’s aortic root aneurysm increased from 45 mm to 58 mm in two years, and a *TRAF7* gene mutation was identified, leading to a Bentall operation. Postoperative imaging findings (Figure 3a–d) revealed improvement in dyspnea symptoms, although desaturation events persisted during sleep. Facial CT (Figure 3e) and laryngoscopy (Figure 3f) revealed narrowing extending from the nasopharynx to the oropharynx. 

**Figure 4:**
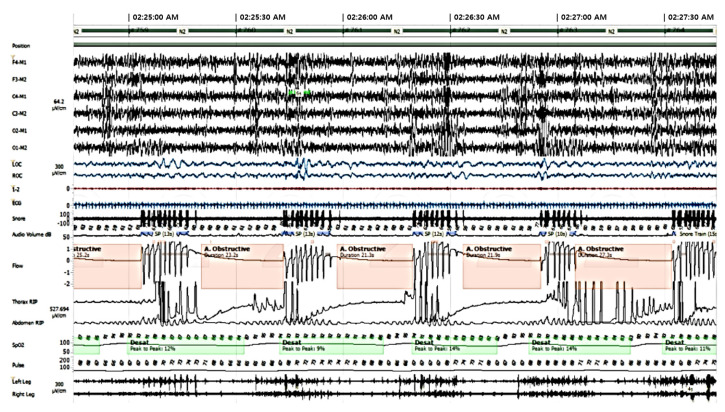
Obstructive sleep apnea on polysomnogram.

Polysomnogram results indicated severe OSA syndrome with an apnea hypopnea index (AHI) of 88/hour, respiratory disturbance index (RDI) of 88/hour, and nadir oxygen saturation of 74% (Figure 4).

## 3. Genetic Diagnosis

**Figure 5:**
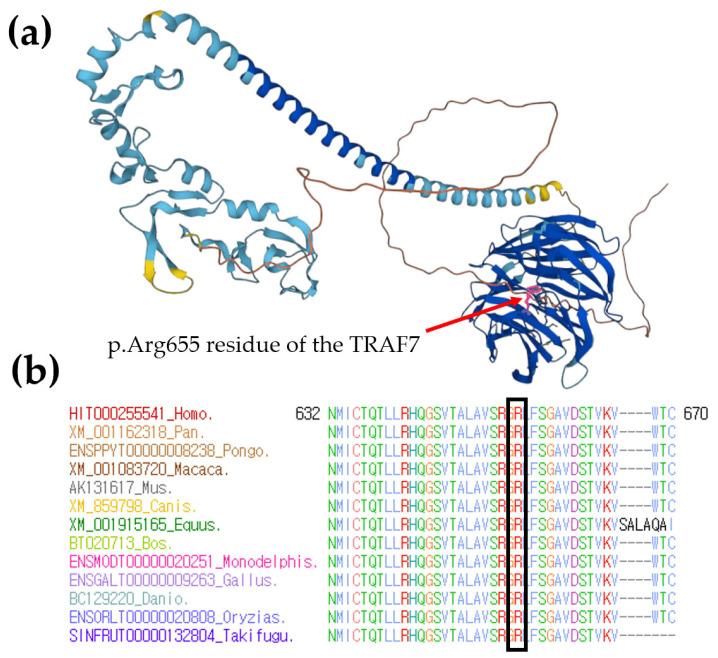
Protein structure and conservation analysis. (**a**) Protein structure analysis using AlphaFold showed very high per-residue confidence scores (pLDDT) of 88.5 for *TRAF7* p.Arg655 residue, highlighted in pink and indicated by a red arrow. (**b**) Sequence alignment of the conserved cytoplasmic domain of the *TRAF7* protein in multiple vertebrate species. Protein sequence of the p.Arg655 residue is highly conserved between all multiple vertebral species. It is highlighted in the empty black box.

To investigate the facial, digital, and cardiac anomalies with developmental delay observed in the proband, we conducted sequential genetic testing targeting various cardiac, facial, and digital anomalies associated with ID. Considering the clinical features of the proband, we suspected connective tissue diseases or Rasopathies. The initial tests included conventional karyotyping and chromosomal microarray analysis, but unfortunately, no pathogenic alterations were identified. Subsequently, we employed a comprehensive clinical exome sequencing (CES) approach using a Celemics G-Mendeliome Clinical Exome Sequencing Panel (Celemics, Inc., Seoul, Republic of Korea). This panel encompasses a wide range of 7000 genes associated with clinically significant Mendelian genetic diseases, including all clinically significant regions. Massively parallel sequencing was conducted using a DNBSEQ-G400RS High-throughput Sequencing Set and DNBSEQ-G400 sequencer (MGI Tech Co. Ltd., Shenzhen, China). The pathogenic variant interpretation followed the standards and guidelines set by the American College of Medical Genetics and Genomics (ACMG) and the Association for Molecular Pathology (AMP). Several variants were identified by CES in the proband with facial, digital, and cardiac anomalies with developmental delay (Supplementary Table S1). Among them, the gene panel sequencing identified a heterozygous *TRAF7* variant, c.1964G>A; p.Arg655Gln, as the best candidate for causing CAFDADD syndrome in the proband (Reference transcript ID: NM_032271.3). The clinical presentation of the patient was partially consistent with the *TRAF7* variant, indicative of facial, digital, and cardiac anomalies with developmental delay. This presentation included developmental delay, congenital anomalies, dysmorphic features, and a wide spectrum of congenital cardiac defects. Sanger sequencing confirmed the segregation of the *TRAF7* c.1964G>A; p.Arg655Gln variant with the phenotype and established the de novo autosomal dominant status of the heterozygous variant in the patient, but not in his parents and sibling. This variant was classified as pathogenic according to ACMG guidelines, considering the following criteria: PS2, PM1, PM2, PP1, PP2, and PP3. Hence, it can be elucidated that the onset of the disease in the patient occurred due to spontaneous mutations. Protein structure analysis using AlphaFold revealed very high per-residue confidence scores (pLDDT) of 88.5 for the TRAF7 p.Arg655 residue (Figure 5a). Sequence alignment of the conserved cytoplasmic domain of the *TRAF7* protein in multiple vertebrate species showed that the protein sequence of the p.Arg655 residue is highly conserved between humans and Takifugu (Figure 5b).

## 4. Discussion

TRAF7 is composed of 21 exons and is located in the chromosomal region 16p13.3. The TRAF7 protein comprises an N-terminal ring finger domain, an adjacent zinc-finger domain, a centrally located coiled-coil motif, and seven WD40 repeats in the C-terminal domain [7]. The majority of pathogenic TRAF7 variants occur in the WD40 repeats, and all of these variants involve highly conserved amino acids, as confirmed by alignments across 100 vertebrates [2,4,8,9,10].

The common phenotypic characteristics associated with TRAF7 mutations include facial dysmorphism (epicanthal folds, ptosis, dysmorphic ear, and blepharophimosis), skeletal deformities (short neck, chest deformity, vertebral abnormalities, and digital deviations), and congenital heart defects (patent ductus arteriosus and valve deformity) [2,4,8,9,10]. Relatively less-frequent features encompass sensorineural hearing loss, developmental delay/intellectual disability, cortical blindness, and abnormal brain images such as cerebral atrophy and prominent ventricles.

The 2021 ESC/EACTS Guidelines for the management of valvular heart disease recommend surgery for aortic root or ascending aortic aneurysms when the maximal ascending aortic diameter exceeds 55 mm, or is greater than 50 mm in patients with Marfan syndrome, or exceeds 45 mm when additional risk factors or gene mutation is present, or exceeds 50 mm when bicuspid valve is present with additional risk factors or coarctation [11]. Aortic aneurysms can arise from various conditions, such as atherosclerosis, inflammation, and vessel injury. However, given the patient’s history of congenital impairments, distinct physical features, and the presence of bicuspid aortic valves, there is suspicion that the patient’s aortic root aneurysm is associated with an underlying genetic disorder such as Marfan syndrome, Ehlers–Danlos syndrome, and Loeys–Dietz syndrome, all of which are inherited connective tissue disorders often linked with aortic aneurysms [1,2].

In the Republic of Korea, the coverage of genetic testing under medical insurance is relatively recent. According to the patient’s mother, the patient attended a school for the blind and, despite being able to read basic Braille, does not exhibit age-appropriate intelligence. Throughout childhood, the patient primarily received ophthalmic care related to blindness, mainly due to economic constraints. Moreover, a breakdown occurred in the relationship between the patient’s mother and the healthcare provider, resulting in the patient being solely cared for by the mother without further medical examinations. During this period, an author with qualifications in pediatric genetic counseling successfully conducted the testing through appropriate explanation and persuasion to the patient and his family. Furthermore, the benefits of utilizing medical insurance coverage for genetic family testing and easing financial burdens were explained, facilitating the progression of genetic testing. As of now, ClinVar has documented 50 *TRAF7* variants, with several carrying pathogenic or likely pathogenic classifications (https://www.ncbi.nlm.nih.gov/clinvar/?term=traf7%5Bgene%5D; accessed on 1 February 2024). These reported cases contribute to our understanding of *TRAF7* variants, showcasing the condition’s variable expressivity within a nuclear family. The detailed clinical characteristics of the reported individuals caused by the *TRAF7* p.Arg655Gln variant are described in Supplementary Table S1 [1,2,3]. Furthermore, the detection of a mosaic variant highlights the potential for a complete CAFDADD syndrome phenotype in mosaic patients who exhibit PDA, ID, ptosis, and dysmorphic features [10]. Our study further expands the understanding of the phenotype associated with *TRAF7*-related neurodevelopmental disorder with multiple anomalies by detailing a patient with a *TRAF7* pathogenic variant who presented a history of intestinal malrotation, a feature not previously reported in the literature. In previous reports, all of the *TRAF7* mutations associated with CAFDADD are missense, and some variants are recurrent with one of the major hotspots (to c.1964G>A; p.Arg655Gln). As our knowledge of disease-causing genes advances, it is crucial to report variants identified in patients from under-represented backgrounds. This practice is essential for future variant interpretation, aiding in a comprehensive understanding of the genetic landscape and ensuring accurate clinical assessments.

Loeys–Dietz syndrome (LDS) is an autosomal-dominant connective tissue disorder resulting from mutations in genes related to the transforming growth factor β signaling pathway (TGFBR1 and TGFBR2). Its phenotype is most similar to that of CAFDADD syndrome. Marfan syndrome (MFS) and Ehlers–Danlos syndrome (EDS) also display overlapping phenotypic characteristics with CAFDADD syndrome, although to a lesser extent than with LDS [1,2]. The common features of LDS include aortic aneurysm, arterial tortuosity, arachnodactyly, pectus deformity, scoliosis, joint laxity, and abnormal uvula [2], which closely resemble those of the reported patient. MFS, an autosomal dominant connective tissue disorder caused by mutations in the fibrillin 1 gene (*FBN1*), often results in cardiovascular abnormalities, with the most common being dilatation of the aortic root [1]. EDS, a group of connective tissue disorders caused by mutations in collagen type V alpha genes (*COL3A1*, *COL5A1*, and *COL5A2*), is inherited through dominant autosomal, recessive autosomal, or x-linked patterns. Aortic root aneurysm is a distinguishing feature of several types of EDS [1]. On the other hand, CAFDADD syndrome is an extremely rare genetic disorder, and this is the first reported case in the Republic of Korea. The patient’s bicuspid valve is attributed to the *TRAF7* variant, which ultimately led to aortic root aneurysm and subsequent heart failure. The dilation of the right ventricle, on the other hand, is unrelated to the cardiac anomaly resulting from the *TRAF7* variant but is rather a consequence of ongoing sleep apnea due to the patient’s spinal anomaly from the *TRAF7* variant. In this case, OSA was revealed, and continuous airway pressure led to improved dyspnea, differing from other cases. Dysmorphic features, including neck hump, short neck, and muscle weakness, may lead to OSA. However, understanding these genetic links can provide valuable insights into the condition’s underlying mechanisms and potentially lead to more personalized approaches to diagnosis and treatment in the future.

## 5. Conclusions

We reported a sporadic case of OSA as a phenotype in patients with CAFDADD syndrome. The identified de novo variant, c.1964G>A; p.Arg655Gln, in *TRAF7*, along with the associated phenotype, contributes to expanding the genetic spectrum of the exceptionally rare CAFDADD syndrome. Furthermore, the presented known c.1964G>A; p.Arg655Gln variant supports previous observations regarding the phenotypic range of *TRF7* germline variants. In individuals exhibiting characteristic dysmorphic features, particularly within the palpebral fissure (blepharophimosis and/or ptosis), congenital heart and skeletal defects, and psychomotor delay, a suspicion of the *TRAF7* variant is warranted. Consistent ophthalmic, neurological, and cardiological assessments, along with early developmental support and motor rehabilitation, play a crucial role in managing patients with the syndrome resulting from *TRAF7* variants. *TRAF7* mutations have been associated with a variety of medical conditions, including potential risks for OSA.

## Data Availability

Data are contained within the article.

## Supplementary Materials

The following supporting information can be downloaded at https://www.mdpi.com/article/10.3390/ijms25073701/s1.
