# Supplementary material for: The First Korean Case with Cardiac, Facial, and Digital Anomalies with Developmental Delay Caused by De Novo TRAF7 p.Arg655Gln Variant
Source: Int J Mol Sci. 2024 Mar 26;25(7):3701. doi: 10.3390/ijms25073701 (PMC11011995; doi:10.3390/ijms25073701)
Supplement: Supplementary file 1 [file ijms-25-03701-s001.zip › ijms-2896151-supplementary.pdf]

Supplementary Table S1. Variants identified by clinical exome sequencing in the proband with facial, digital, cardiac anomalies with developmental delay

Gene	Reference transcript ID	Base change	Codon change	Variant type	OMIM phenotype	Inheritance
ABCA7	NM_019112.4	c.5961G>T	p.Glu1987Asp	missense	{Alzheimer disease 9, susceptibility to}	AD
AKT2	NM_001626	.6c.431G>A	p.Arg144Gln	missense	Diabetes mellitus, type II ∣ Hypoinsulinemic hypoglycemia with hemihypertrophy	AD ∣ AD
ANK3	NM_020987.	5c.8506C>T	p.His2836Tyr	missense	Intellectual developmental disorder, autosomal recessive 37	AR
DNAH10	NM_207437.3	c.10444C>T	p.Arg3482Cys	missense	Spermatogenic failure 56	AR
FSIP2	NM_173651.4	c.3351G>A	p.Met1117Ile	missense	Spermatogenic failure 34	AR
HSPA9	NM_004134.7	c.661A>C	p.Asn221His	missense	Anemia, sideroblastic, 4 ∣ Even-plus syndrome	AD ∣ AR
HYDIN	NM_001270974.2	c.13080G>T	p.Glu4360Asp	missense	Ciliary dyskinesia, primary, 5	AR
IGSF3	NM_001542.4	c.1784G>A	p.Trp595Ter	nonsense	?Lacrimal duct defect	AR
ITGA8	NM_003638.3	c.3128G>T	p.Arg1043Ile	missense	Renal hypodysplasia/aplasia 1	AR
LIPA	NM_000235.4	c.10C>T	p.Arg4Trp	missense	Cholesteryl ester storage disease ∣ Wolman disease	AR ∣ AR
RECQL	NM_002907.4	c.1784A>G	p.Gln595Arg	missense	RECON progeroid syndrome	AR
TPP2	NM_003291.4	c.1067A>G	p.Tyr356Cys	missense	Immunodeficiency 78 with autoimmunity and developmental delay	AR
TRAF7	NM_032271.3	c.1964G>A	p.Arg655Gln	missense	Cardiac, facial, and digital anomalies with developmental delay	AD
VANGL2	NM_020335.3	c.1319G>A	p.Arg440Gln	missense	Neural tube defects	AD

OMIM, Online Mendelian Inheritance in Man; AD, autosomal dominant; AR, autosomal recessive
